# Role of Acupoint Area Collagen Fibers in Anti-Inflammation of Acupuncture Lifting and Thrusting Manipulation

**DOI:** 10.1155/2017/2813437

**Published:** 2017-04-04

**Authors:** Fan Wang, Guang-wei Cui, Le Kuai, Jian-min Xu, Ting-ting Zhang, Huai-jin Cheng, Hong-sheng Dong, Gui-rong Dong

**Affiliations:** ^1^School of Acupuncture-Moxibustion and Tuina, Shanghai University of Traditional Chinese Medicine, Shanghai, China; ^2^Shanghai Research Institute of Acupuncture and Meridian, Shanghai, China; ^3^Department of Acupuncture and Moxibustion, Yueyang Hospital of Integrated Traditional Chinese and Western Medicine, Shanghai University of Traditional Chinese Medicine, Shanghai, China

## Abstract

The role of the acupoint area collagen fibers in the efficacy of acupuncture lifting and thrusting (L&T) manipulation will be explored in this paper. 30 male NZW rabbits were randomly divided into 6 groups: sham operation group (Group N), model group (Group M), acupuncture without manipulation group (Group W), acupuncture L&T manipulation group (Group A), collagenase pretreatment group (Group JM), and collagenase pretreatment + acupuncture L&T manipulation group (Group JA). The bacterial endotoxin was used to generate the rabbit fever models. Acupuncture was applied at IL-11. The levels of IL-1*β*, TNF-*α*, and IL-4 and the rectal temperature were measured at 2 h, 4 h, and 6 h after modeling and the collagen fiber morphology at acupoint area was observed after 6 hours.* Results.* As compared with Group N, the levels of IL-1*β* and TNF-*α* in Group M were significantly higher; the level of IL-4 was significantly lower (*P* < 0.05). As compared with Group M, IL-1*β* and TNF-*α* in Groups W and A were significantly lower and IL-4 was significantly higher (*P* < 0.05). As compared with Group W, IL-1*β* and TNF-*α* in Group A were lower and IL-4 was higher (*P* < 0.05). The collagen fiber in Group A was slightly rough, distorted, and fractured. As shown in studies, the endotoxin-induced inflammatory response can be significantly inhibited by acupuncture whose efficacy can also be significantly improved by the manipulations. Collagenase pretreatment may be the first receptor to the mechanical force of the L&T manipulation.

## 1. Introduction

Acupuncture manipulation is the operation of the filiform needle after insertion to enhance the acupuncture feeling or spread such feeling towards certain direction [1]. The lifting and thrusting method is one of the most widely used manipulations in clinical practice [2]. For the ancients said “Qi arrival is the key to acupuncture efficacy” (“Lingshu: Nine Needles and Twelve Primary Points”), the Chinese medicine considers acupuncture manipulation as the key factor for Qi conversation, adjustment, and “arrival” [3, 4]. Therefore, to improve the clinical curative effect, it is of great significance to clarify the mechanisms and rules of acupuncture manipulation.

In the research of the mechanism of acupuncture manipulation efficacy, it is the core issue to find how the mechanical stress produced by acupuncture manipulation is perceived by the body and transformed into the biological signals. Aiming at clarifying this mystery, more and more researchers shift their attention to the fascia and connective tissue. Acupoint anatomy and other studies have found that the body's meridians, acupoints, and connective tissue are closely interconnected [5, 6]. Collagen fiber is an important part of the connective tissue. Studies have also found that, after lifting, thrusting, and twisting at the acupoints, the residues warped on the pulled needles are mainly collagen fibers [7]. The morphology of the collagen fibers at the acupoint area can be influenced differently with the filiform needles of different sizes [8]. Subtle differences in bidirectional acupuncture needle rotation techniques can affect cellular responses in mouse subcutaneous connective tissue [9, 10]. Is there correlation between the collagen fiber morphological changes at the acupoint areas and the acupuncture effect? Will the acupuncture effect be influenced after destroying the collagen fibers at the acupoint areas? Rare researches have been reported on these questions.

In this study, we tried to observe whether the acupoint area collagen fibers are involved in the process of converting the mechanical signal of the acupuncture to biological signal. The bacterial endotoxin was used to generate the rabbit fever models in this study which were treated with acupuncture at “Quchi” acupoint along with the lifting and thrusting (L&T) manipulation for intervention during retention. The bacterial endotoxin will act on the mononuclear macrophages and neutrophils, producing and releasing IL-1*β*, TNF-*α*, and other inflammatory factors, while generating anti-inflammatory factor IL-4 for immunosuppression. Inflammatory factors IL-1*β* and TNF-*α* as the fever messengers can directly or indirectly act on the thermoregulation center and cause fever [11]. Therefore, in this study, the levels of IL-1*β*, TNF-*α*, and IL-4 in the serum were used as the primary endpoints with the rabbit rectal temperature as a secondary endpoint. In addition, the morphological changes of the collagen fibers at the acupoint areas were also used as a secondary endpoint. The details are as follows.

## 2. Materials and Methods

### 2.1. Materials

*(1) Animals.* A total of 30 healthy male New Zealand White (NZW) rabbits, weighing 2–2.2 kg, were provided by the Experimental Animal Center of Shanghai University of Traditional Chinese Medicine [license number: SXCK (Shanghai) 2012-0008]. Basal body temperature (BBT) screening was conducted prior to the experiment.

*(2) Major Agent.* Bacterial endotoxin (Sigma, L2630, 10 mg), type I collagenase (Sigma, lot number: C0130), Masson staining kit (Beijing Reagan, lot number: DC0032), rabbit TNF-*α* kit (R&D Systems subpackage, rb201510200842), IL-1*β* kit (R&D Systems subpackage, rb201510190821), and IL-4 kit (R&D Systems subpackage, rb201510270951) were the major agents used in this study.

*(3) Major Instruments.* Pyrogen measurer (ZRY-3, Tianjin Tianda Tianfa), Upright Microscope (CX41, OLYMPUS), IMS Image Analysis System (Jrdun Biotechnology, Shanghai), Microplate Reader, and a spectrophotometer were used in this study.

### 2.2. Method

#### 2.2.1. Environmental Adaptation and Grouping

All NZW rabbits were reared and used in the same standard environment, with natural lighting, natural drinking, and dieting. Three hours before the experiment, rectal temperature of NZW rabbits was measured once per hour with the BBT calculated as the average of the three measurements. The NZW rabbits with the BBT beyond 38.5 ± 0.5°C were excluded. The 30 male NZW rabbits were randomly divided into 6 groups (5 per group), that is, sham operation group (N), model group (M), acupuncture without manipulation group (W), acupuncture manipulation group (A), collagenase pretreatment group (JM), and collagenase pretreatment group + acupuncture manipulation group (JA). The interventions conducted for each group were as follows.

*Sham Operation Group (N).* This group was injected with 50 *μ*L saline at bilateral “Quchi” acupoint areas and, 30 min later, injected with saline at the dosage of 1 mL/kg at the rabbit ear vein without endotoxin injection or acupuncture.

*Model Group (M).* This group was injected with 50 *μ*L saline at bilateral “Quchi” acupoint areas and, 30 min later, injected with 3 *μ*g/mL bacterial endotoxin at the dosage of 1 mL/kg at the rabbit ear vein to establish the fever model without any treatment.

*Acupuncture without Manipulation Group (W).* We followed the same protocol as in the model group but 1.5 hours after modeling we applied acupuncture at bilateral “Quchi” acupoint areas without manipulation.

*Acupunctural Manipulation Group (A).* We followed the same protocol as in the model group but 1.5 hours after modeling we applied acupuncture with L&T manipulation at bilateral “Quchi” acupoint areas.

*Collagenase Pretreatment Group (JM).* This group was injected with 2 mg/mL type I collagenase of 50 *μ*L at bilateral “Quchi” acupoint areas to destroy the underneath collagen fibers, followed, 30 min later, by an injection of 3 *μ*g/mL bacterial endotoxin at the dosage of 1 mL/kg at the rabbit ear vein to establish the fever model without any treatment.

*Collagenase Pretreatment + Acupuncture Manipulation Group (JA).* We followed the same protocol as in Group JM but 1.5 hours after modeling we applied acupuncture with L&T manipulation at bilateral “Quchi” acupoints.

#### 2.2.2. Acupuncture at the “Quchi” Point

Refer to Schedule 3 “Commonly Used Acupoints of Rabbit” in Experimental Acupuncture [12] for the “Quchi” acupoint localization on NZW rabbits (front lateral depression of the elbow). Prior to the experiment, the body hair was removed. The *ϕ*0.25 mm × 25 mm filiform needle was used for acupuncture at the bilateral “Quchi” points with the depth about 10 mm. The needle was removed for 30 min.

*L&T Manipulation.* The L&T was conducted with the amplitude of about 2 mm and the frequency of 60 cycles/min. Each cycle lasted 30 s at insertion, 10 min, and 20 min later.

**Figure 1:**
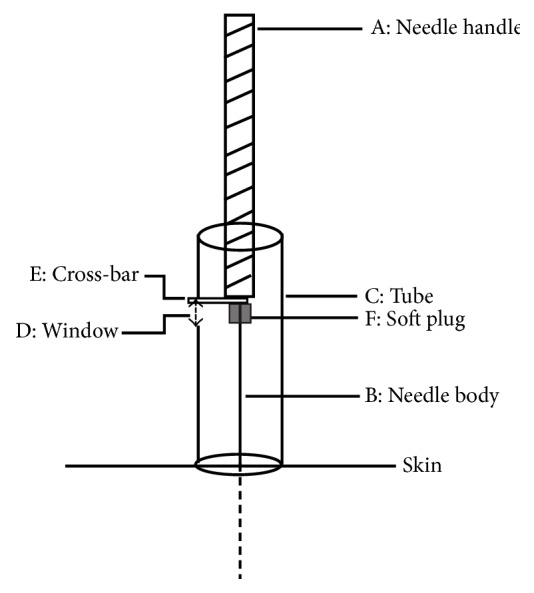
Self-made controller. B: needle body. The full line is the exposed part, while the imaginary line is the inserted part. C: tube, which is 1 cm long and cut from needle tube. D: window on the tube, 2 mm long, which is just the amplitude of lifting-thrusting manipulation. E: cross-bar, which is made of cardboard. F: soft plug, which is used to prevent cross-bar falling down.

Acupuncture was performed by a single skilled acupuncturist. During the process of acupuncture, the self-made tube (see Figure 1) was used to control the amplitude and the metronome was used to control the frequency.

#### 2.2.3. Endpoints and Methodology

(1) Collection and processing of the blood sample and measurement of each endpoint: the remaining needles were inserted into the median artery of rabbit ear for collecting the blood samples at 2 h, 4 h, and 6 h after modeling, about 2 mL each time. The blood was left to stand for a few minutes before centrifugation for 10 min under 3000 r·min^−1^. The separated serum was placed in the refrigerator under −80°C. The levels of the serum inflammatory factors, TNF-*α*, IL-1*β*, and IL-4, were determined by enzyme-linked immunosorbent assay (ELISA). All the tests were carried out in strict accordance with the INSTRUCTIONS FOR USE on the kit packages.
(2) Measurement and recording of the rectal temperature: the rectal temperatures of the NZW rabbits were recorded once at 2 h, 4 h, and 6 h after modeling, respectively, and the rate of rectal temperature rise (Δ*T*%) was calculated. *T* was the immediate rectal temperature, and *T*_0_ was the BBT:
  (1) $$\begin{array}{c} \Delta T\%=\frac{\left(T-T_{0}\right)}{T_{0}}. \end{array}$$
(3) After temperature measurements: the NZW rabbits were sacrificed immediately through air embolism. For the sham operation group (N), acupuncture manipulation group (A), collagenase group (JM), and collagenase + acupuncture manipulation group (JA), the subcutaneous tissue with the volume of 15 × 15 × 3 (mm^3^) at the left “Quchi” acupoint area was taken and fixed in 10% formalin for 48 h. The tissue was then dehydrated, embedded in the paraffin, and sliced in series (with the thickness of 5 *μ*m) in sagittal plane of the tissue blocks. The slices were then dewaxed, stained with Masson method, conventionally dehydrated, transparentized, and mounted. The morphology of the collagen fibers in the subcutaneous tissue sections was observed under 200x fold light microscope.

#### 2.2.4. Statistical Analysis

SPSS 21.0 software was used for statistical analysis, with *P* < 0.05 considered as statistically significant, and two-tailed test was conducted. Only measurement data were used. The data were normally distributed, and the statistical results were described with $\overset{-}{x}\pm s$. Between-group comparisons were conducted using variance analysis of the repeated measurements. For the data of the same time point, between-group comparisons were conducted with one-way ANOVA and for multiple comparisons, the SNK tests were conducted.

## 3. Results and Analysis

### 3.1. Effects of Acupuncture on Endogenous Pyrogen (EP) in NZW Rabbits with Endotoxin-Induced Fever

The variance analysis of the repeated measurements indicated that the significant between-group differences were found in the levels of serum IL-1*β* and serum TNF-*α* for the time points of 2 hours, 4 hours, and 6 hours after modeling (*F* = 361.792, *P* = 0.000; *F* = 100.475 *P* = 0.000). There was no temporal regularity for the variance of the serum IL-1*β* levels (*F* = 0.250, *P* = 0.622), but the serum TNF-*α* levels decreased with time (*F* = 6.920, *P* = 0.015). It can be seen from Tables 1 and 2 that, compared with Group N, the levels of IL-1*β* and TNF-*α* in Group M were significantly higher at 2 hours, 4 hours, and 6 hours after modeling (*P* < 0.01) and that, compared with Group M, Groups W and A had significantly lower rate of increment (*P* < 0.01) wherein Group A was significantly lower than Group W (*P* < 0.01). It is suggested that the acupuncture could significantly inhibit the level increment of serum IL-1*β* and TNF-*α* induced by endotoxin and that the acupuncture manipulation could significantly improve the curative effect of acupuncture.

The destroyed regional collagen fiber with collagenase at the “Quchi” acupoint had no effect on the levels of serum IL-1*β* and TNF-*α*, and there was no significant difference compared with Group M (*P* > 0.05). The levels of serum IL-1*β* and TNF-*α* in Group JA were significantly higher than those of Group A (*P* < 0.05) but had no significant difference compared with Group W (*P* > 0.05). It is suggested that collagenase pretreatment significantly affected the acupuncture manipulation efficacy and that the collagen fibers at the acupoint area may be an important link in the reception of the mechanical stimulation of acupuncture.

### 3.2. Effects of Acupuncture on Anti-Inflammatory Factor IL-4 in NZW Rabbits with Endotoxin-Induced Fever

The variance analysis of the repeated measurements indicated that the serum levels of IL-4 were significantly different among the groups at 2 h, 4 h, and 6 h after modeling (*F* = 203.499, *P* = 0.000). There was no temporal regularity for the variance of the serum IL-1*β* levels (*F* = 0.060, *P* = 0.808). It can be seen from Table 3 that, compared with Group N, the levels of serum IL-4 in Group M were significantly lower at 2 h, 4 h, and 6 h after modeling (*P* < 0.01) and that, compared with Group M, Groups W and A had significantly higher levels (*P* < 0.01) wherein Group A had significantly higher levels than Group W (*P* < 0.01). It is suggested that the acupuncture could significantly increase the level of serum anti-inflammatory factor IL-4 and that the acupuncture manipulation could significantly improve the curative effect of acupuncture.

The destroyed regional collagen fiber with collagenase at the “Quchi” acupoint had no effect on the levels of serum IL-4, and there was no significant difference compared with Group M (*P* > 0.05). The levels of serum IL-4 in Group JA were significantly lower than those of Group A (*P* < 0.01) but had no significant difference compared with Group W (*P* > 0.05). It is suggested that collagenase pretreatment significantly affected the acupuncture manipulation efficacy and that the collagen fibers at the acupoint area may be an important link in the reception of the mechanical stimulation of acupuncture.

### 3.3. Effect of Acupuncture on the Rectal Temperature in NZW Rabbits with Endotoxin-Induced Fever

The variance analysis of repeated measurements indicated that there was significant difference among the groups at 2 h, 4 h, and 6 h after modeling (*F* = 1291.230, *P* = 0.000) and that the change of rectal temperature was related to time (*F* = 144.928, *P* = 0.000) with the peak appearing at 4 h after modeling. As can be seen from Table 4, the rate of rectal temperature rise in Groups W and A was significantly lower than that in Group M at 2 h, 4 h, and 6 h after modeling (*P* < 0.01), and Group A had significantly lower rate than Group W (*P* < 0.05). It is suggested that the acupuncture could significantly suppress the fever caused by the endotoxin, the effect of acupuncture could be obvious and swift, and the effect of cooling could still be significant at 4 h after acupuncture. The acupuncture manipulation could significantly improve the curative effect of acupuncture.

The destroyed regional collagen fiber with collagenase at the “Quchi” acupoint had no effect on the rectal temperature, and there was no significant difference compared with Group M (*P* > 0.05). The rate of rectal temperature rise at different interval in Group JA was significantly higher than that of Group A (*P* < 0.05) but had no significant difference compared with Group W (*P* > 0.05). It is suggested that collagenase pretreatment significantly affected the acupuncture manipulation efficacy and that the collagen fibers at the acupoint area may be an important link in the reception of the mechanical stimulation of acupuncture.

### 3.4. Morphological Observation of the Collagen Fibers at Acupoint Areas

**Figure 2:**
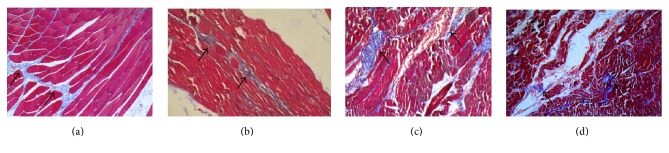
Masson staining microscopic picture of the subcutaneous tissue taken from the Quchi acupoint. Collagen fiber appears blue (arrow), while muscle tissue and blood cell appear red. (a) Group M. (b) Group A. (c) Group JM. (d) Group JA.

Under the light microscope, it is found that the muscle and collagen fibers at the Quchi acupoint area in Group N were arranged in bundles in regular direction, and the collagen fibers were curled. The surface of collagen fiber was slightly rough in Group A with twisted muscle tissue and collagen fibers accompanied with partial fracture. After collagenase pretreatment, the muscle tissue structure was disordered and mixed with the destroyed collagen fibers. The blood vessels were also damaged with red blood cells being released into the interstitial space. Group JA is similar to Group JM (see Figure 2).

## 4. Discussion

This study was the first to analyze the role of collagen fibers in the efficacy of acupuncture manipulation through injecting collagenase at the “Quchi” acupoints of the NZW rabbits' fever models. It provides the basis for the hypothesis that the effect mechanism of acupuncture manipulation may relay on the collagen fibers to transmit the acupuncture signals to the peripheral cells.

In this study, we observed that the levels of serum inflammatory factors IL-1*β* and IL-6 began to increase immediately after modeling, peaked at 2 h, and remained stable from 2 h to 6 h. The BBTs of the NZW rabbits began to increase significantly about 1 h after modeling, lasted for almost 5 hours, and peaked at 4 h. For acupuncture at Quchi acupoints with filiform needles with or without manipulation, the serum inflammatory factors IL-1*β* and IL-6 in NZW rabbits with fever can be decreased and the level of anti-inflammatory factor IL-4 can be increased while the rectal temperature can be lowered. “Quchi” acupoint is the He sea-point of Large Intestine Meridian of Hand, Yangming. In clinical practice, Quchi is used as the primary acupoint to treat fever [13], acne [14], allergic eczema [15], and other pyrexia or inflammations in the face [16]. There is a study that indicates that the acupuncture at Quchi-Hegu can lower the temperature of the rats with fever by reducing the level of central pyrogenic agent PGE2 in hypothalamus [17]. In this study, the temperature of the rabbits with fever was lowered by acupuncture treatment, which is consistent with the result of the aforementioned study. Besides, the results supplement the peripheral mechanisms of the anti-inflammatory effect of the acupuncture at Quchi acupoint.

This study also found that, for the benign regulation of the levels of IL-1*β*, IL-6, IL-4, and rectal temperature, the acupuncture with lifting and thrusting manipulation group shows better results than the acupuncture without manipulation group. At the same time, the morphology of the regional collagen fibers at the acupoint areas was changed due to the intervention of L&T manipulation: the surface of collagen fiber was slightly rough with twisted muscle tissue and collagen fibers accompanied with partial fracture. Is there any linkage between the acupuncture manipulation and the morphological changes of the collagen fibers caused by it? For this purpose, the collagenase group was arranged in the experiment in which the acupoint areas were given collagenase pretreatment before modeling. Under the light microscope, after collagenase pretreatment, part of the muscle tissue structure was disordered and mixed with the destroyed collagen fibers. The blood vessels were also damaged with red blood cells being released into the interstitial space. This indicated that the collagenase concentration and dosage used in this study were enough to destroy the acupoint area collagen fibers. There was no statistically significant difference in terms of the levels of serum IL-1*β*, IL-6, and IL-4 between the collagenase pretreatment group and the model group, indicating that regional injection of collagenase at the acupoint area had no significant effect on the levels of the serum cytokines. The levels of serum cytokines of Groups JA and A were comparable. Compared with Group M and Group W, the levels of serum IL-1*β*, IL-6, and IL-4 and the rectal temperature in Group JA did not show significant difference, but compared with Group A, they did. This suggested the correlation between the regional collagen fibers at the acupoint area and the efficacy of the acupuncture manipulation.

Collagen fibers are mainly composed of the collagen protein and arranged in bundle or reticular form. It is the main protein composition in the extracellular matrix of the connective tissue. From the perspective of biomechanics, collagen is a three-dimensional long-range ordered structure, with the nature of liquid crystalline continuum. It sensitively responds to the minor change of the environment [18]. The research group led by Langevin believes that the acupuncture effect may be explained as that the manipulation of lifting, thrusting, and twisting the needle inserted into the acupoint causes the nearby collagen fibers to be twisted and wound, by way of which the mechanical force signals pass on to the connective tissue cells and even spread further [9, 19–21]. As observed by Julias et al. [22], the manipulation of twisting the needle led to different extents of parallelism of the collagen fiber bundles. It is believed that the force signals pass on to the connective tissue cells via the deformation of the collagen fibers in the extracellular matrix. As found by Yu et al. [23], the destruction of the collagen structure at Zusanli acupoint area of AA rat with type I collagenase significantly inhibited the degranulation of the mast cells at this acupoint area, and the manipulation of lifting, thrusting, and twisting showed significantly weakened analgesic effect. These studies indicate that the collagen fibers at the acupoint area are the first receptors of the acupuncture mechanical force. They receive the acupuncture mechanical signals and pass on to the periphery to stimulate the cell-level signal transduction. However, most of the previous studies focused on the morphological changes of collagen fiber before and after acupuncture, while this study observed the collagen fiber and the effect of acupuncture simultaneously. After the collagen fibers were destroyed, the mechanical force signals of the manipulation of lifting and thrusting could not be accepted or further transformed into biological signals. Therefore, the anti-inflammatory effect of acupuncture was reduced. The correlation between collagen fibers in acupoint tissues and acupuncture effect is proved in this study.

Generally, the endotoxin-induced inflammatory response can be significantly inhibited by acupuncture whose efficacy can also be significantly improved by the acupuncture manipulation. Collagenase pretreatment will affect the efficacy of the acupuncture manipulation and the acupoint area collagen fibers may be the first receptor to the mechanical force of the L&T manipulation. This study still has certain limitation. Firstly, Group JM and Group JA have to receive collagenase injection in the LI11 area. The syringe needle is inserted from 1 cm in front of LI11 obliquely pointing to LI11. There is no obvious influence on acupoint tissue according to histomorphology observation. However, the effect of syringe needle intervention on acupuncture is inevitable. To furthest reduce deviation due to needle intervene, all the other groups are injected with saline. Secondly, the change of connective tissue extracellular matrix caused by acupuncture manipulation does not directly produce biological effects. This change will lead to the degranulation of the mast cells or the change of the morphology and secretory function of the fibroblasts. It shall pass the cascade of stimulating-initiating-information transmitting-amplifying before acting on the target organ [24–26]. In this study, only the role of collagen fibers in this process was observed and there is no observation regarding the morphology or function of the effector cells (the fibroblasts and mast cells, etc.) or their relationship with the collagen fibers. However, this will be the future research direction of our research group.

## Figures and Tables

**Table 1:** Effect of acupuncture on the level of serum IL-1*β* in NZWs with endotoxin-induced fever ($\overset{-}{x}\pm s$, in ng/L).

Group	*N*	2 h	4 h	6 h
N	5	22.69 ± 2.23	21.72 ± 2.54	22.32 ± 1.88
M	5	42.35 ± 0.86^a^	43.57 ± 2.01^a^	42.80 ± 1.44^a^
W	5	38.9 ± 0.61^ab^	38.24 ± 1.87^ab^	39.15 ± 2.74^aB^
A	5	30.00 ± 0.98^abc^	28.66 ± 2.44^abc^	27.52 ± 2.58^abc^
JM	5	42.95 ± 1.48^ad^	43.26 ± 0.42^acd^	42.33 ± 1.72^aCd^
JA	5	41.08 ± 1.55^ad^	39.51 ± 1.37^abd^	41.65 ± 2.40^ad^
*F*		170.529	105.098	81.531
*P*		0.000	0.000	0.000

Note: ^a^compared with Group N, *P* < 0.01; ^b^compared with Group M, *P* < 0.05; ^B^compared with Group M, *P* < 0.05; ^c^compared with Group W, *P* < 0.01; ^C^Compared with Group JM, *P* < 0.05; ^d^compared with Group A, *P* < 0.01. N: sham operation group; M: model group; W: acupuncture without manipulation group; A: acupuncture manipulation group; JM: collagenase pretreatment group; JA: collagenase pretreatment + acupuncture manipulation group.

**Table 2:** Effect of acupuncture on the level of serum TNF-*α* in NZWs with endotoxin-induced fever ($\overset{-}{x}\pm s$, in ng/L).

Group	*N*	2 h	4 h	6 h
N	5	6.04 ± 0.81	5.37 ± 0.15	5.59 ± 0.62
M	5	11.25 ± 0.56^a^	11.60 ± 0.71^a^	11.52 ± 0.64^a^
W	5	10.38 ± 1.01^a^	10.07 ± 0.47^ab^	9.97 ± 0.19^ab^
A	5	8.43 ± 1.80^abc^	7.28 ± 1.40^abc^	6.65 ± 0.58^abc^
JM	5	11.60 ± 0.42^ad^	11.58 ± 0.69^acd^	11.52 ± 0.35^acd^
JA	5	10.36 ± 0.83^ad^	10.36 ± 0.45^aBdE^	9.80 ± 0.45^abde^
*F*		21.549	56.156	124.656
*P*		0.000	0.000	0.000

Note: ^a^compared with Group N, *P* < 0.01; ^b^compared with Group M, *P* < 0.05; ^B^compared with Group M, *P* < 0.05; ^c^compared with Group W, *P* < 0.01; ^d^compared with Group A, *P* < 0.01; ^e^compared with Group JM, *P* < 0.01; ^E^compared with Group JM, *P* < 0.05. N: sham operation group; M: model group; W: acupuncture without manipulation group; A: acupuncture manipulation group; JM: collagenase pretreatment group; JA: collagenase pretreatment + acupuncture manipulation group.

**Table 3:** Effects of acupuncture on the level of serum IL-4 in NZWs with endotoxin-induced fever ($\overset{-}{x}\pm s$, in ng/L).

Group	*N*	2 h	4 h	6 h
N	5	68.61 ± 4.14	67.72 ± 2.88	67.79 ± 3.69
M	5	34.01 ± 2.25^a^	39.49 ± 3.00^a^	36.81 ± 3.58^a^
W	5	43.25 ± 2.56^ab^	44.00 ± 2.02^ab^	39.85 ± 2.66^ab^
A	5	60.22 ± 3.85^abc^	64.92 ± 3.40^abc^	60.23 ± 3.03^abc^
JM	5	35.24 ± 3.39^acd^	40.68 ± 1.98^acd^	36.56 ± 2.14^acd^
JA	5	43.95 ± 2.87^abd^	44.58 ± 2.54^aBdE^	42.86 ± 3.56^abde^
*F*		91.907	110.480	88.834
*P*		0.000	0.000	0.000

Note: ^a^compared with Group N, *P* < 0.01; ^b^compared with Group M, *P* < 0.05; ^B^compared with Group M, *P* < 0.05; ^c^compared with Group W, *P* < 0.01; ^d^compared with Group A, *P* < 0.01; ^e^compared with Group JM, *P* < 0.01; ^E^compared with Group JM, *P* < 0.05. N: sham operation group; M: model group; W: acupuncture without manipulation group; A: acupuncture manipulation group; JM: collagenase pretreatment group; JA: collagenase pretreatment + acupuncture manipulation group.

**Table 4:** Effect of acupuncture on the rate of rectal temperature rise (Δ*T*%) in NZWs with endotoxin-induced fever ($\overset{-}{x}\pm s$, in %).

Group	*N*	2 h	4 h	6 h
N	5	0.36 ± 0.40	0.36 ± 0.47	−0.05 ± 0.62
M	5	4.68 ± 0.57^a^	6.39 ± 0.91^a^	5.09 ± 0.62^a^
W	5	3.53 ± 0.55^ab^	5.77 ± 0.83^aB^	3.32 ± 0.81^ab^
A	5	2.44 ± 1.06^abC^	4.10 ± 0.69^abc^	1.77 ± 0.60^abC^
JM	5	4.46 ± 0.22^aCd^	6.91 ± 0.70^ad^	4.67 ± 0.60^aCd^
JA	5	3.47 ± 0.60^abDE^	5.02 ± 0.52^abDe^	3.21 ± 0.66^abde^
*F*		32.478	56.689	42.038
*t*		0	0	0

Note: ^a^compared with Group N, *P* < 0.01; ^b^compared with Group M, *P* < 0.05; ^c^compared with Group W, *P* < 0.01; ^C^compared with Group W, *P* < 0.05; ^d^compared with Group A, *P* < 0.01; ^D^compared with Group A, *P* < 0.05; ^e^compared with Group JM, *P* < 0.01; ^E^compared with Group JM, *P* < 0.05. N: sham operation group; M: model group; W: acupuncture without manipulation group; A: acupuncture manipulation group; JM: collagenase pretreatment group; JA: collagenase pretreatment + acupuncture manipulation group.
